# Donor–Recipient Non-HLA Variants, Mismatches and Renal Allograft Outcomes: Evolving Paradigms

**DOI:** 10.3389/fimmu.2022.822353

**Published:** 2022-04-01

**Authors:** Priyanka Jethwani, Arundati Rao, Laurine Bow, Madhav C. Menon

**Affiliations:** ^1^ Department of Medicine, Yale University School of Medicine, New Haven, CT, United States; ^2^ Department of Surgery, Yale University School of Medicine, New Haven, CT, United States

**Keywords:** APOL1, LIMS1, non-HLA variants, donor–recipient mismatches, renal allograft outcomes

## Abstract

Despite significant improvement in the rates of acute allograft rejection, proportionate improvements in kidney allograft longevity have not been realized, and are a source of intense research efforts. Emerging translational data and natural history studies suggest a role for anti-donor immune mechanisms in a majority of cases of allograft loss without patient death, even when overt evidence of acute rejection is not identified. At the level of the donor and recipient genome, differences in highly polymorphic HLA genes are routinely evaluated between donor and recipient pairs as part of organ allocation process, and utilized for patient-tailored induction and maintenance immunosuppression. However, a growing body of data have characterized specific variants in donor and recipient genes, outside of HLA loci, that induce phenotypic changes in donor organs or the recipient immune system, impacting transplant outcomes. Newer mechanisms for “mismatches” in these non-HLA loci have also been proposed during donor–recipient genome interactions with transplantation. Here, we review important recent data evaluating the role of non-HLA genetic loci and genome-wide donor-recipient mismatches in kidney allograft outcomes.

## Introduction

Kidney transplantation is the preferred treatment for patients with end stage kidney disease owing to the survival advantage it confers, compared to maintenance dialysis. Despite success in reducing acute rejection episodes, long term allograft survival has remained an elusive goal and a major research focus for the community (1, 2). While distinct etiologies are identifiable in half of all late allograft loss, allograft fibrosis or interstitial fibrosis and tubular atrophy (IF/TA) of unclear etiology has accounted for 30–40% of cases (3, 4). Even in these cases of IF/TA (without rejection), biopsy transcriptome data has implicated a role for anti-donor immune responses (5). Thus, a majority of allograft loss is related to chronic immune injury.

It is well-known that donor–recipient (D–R) genetic mismatches at the human leukocyte antigen (HLA) region are directly associated with acute renal allograft rejection. For this reason, the organ allocation process has been geared towards precise typing of donor and recipient HLA for organ allocation. In conjunction with patient-tailored induction and maintenance immunosuppression based partly on HLA-matching, this has led to a dramatic improvement in episodes of acute rejection and overall short-term allograft outcomes. However, instances of acute antibody-mediated rejection (AMR) still occur in well-matched and HLA-identical transplants (6, 7). In fact, an evaluation of the UNOS Registry graft survival data in 2003 indicated that only 18% of grafts lost at 10 years could be attributed to HLA-mismatches whereas 38% graft failures were due to immunological reactions against non-HLA factors as seen in HLA-identical sibling grafts (8). Furthermore, only a 15% survival difference exists at 10 years post-transplantation between the fully matched kidneys and the kidneys mismatched for both alleles at the HLA-A, B and DR loci (9) indicating that additional factors influence allograft survival in the modern context, namely, both genetic and environmental factors. Among genetic influences on allograft survival, these data suggest donor- and recipient-genetic loci outside of usually typed HLA regions that induce or modulate anti-donor responses and impact long-term graft survival. In this paper, we aim to review available data evaluating the role of non-HLA genetic loci and genome-wide D–R interactions in kidney allograft outcomes.

## Non-HLA Genetic Variants and Proposed Mechanisms

In the traditional paradigm, alloimmune responses that manifest as allograft rejection arise from T‐lymphocytic “non‐self” recognition, when recipient T cells recognize donor antigens *via* the direct pathway (donor major histocompatibility complex [MHC] plus peptide on donor cells), indirect pathway (donor‐derived antigens presented by recipient antigen presenting cells [APC]), or the semidirect pathway (presentation of self‐peptides by donor MHC on recipient APC *via* membrane transfer). Fundamentally, alloreactivity (i.e., anti-donor response in organ transplantation) is based on specific peptide/MHC differences between the host (recipient) and donor cells giving rise to a classical adaptive immune response. At the level of the genome, the processes that recognize the donor organ as non-self and result in acute organ rejection (AR) are determined by differences in the human leukocyte antigen (HLA) region between the donor– and recipient (D–R) pair or HLA-mismatches. Indeed, AR itself has been repeatedly shown to be associated with decreased allograft survival (10, 11). However, elegant mechanistic data proposed HLA-independent loci and demonstrated mechanisms outside of T-lymphocytic anti donor responses (12, 13) in experimental transplant models. Further, several translational genetic association studies demonstrate a role for non-HLA loci in AR and transplant outcomes (10, 14–16).

Each donor–recipient (D–R) pair of genomes contains vast permutations of non-synonymous amino-acid differences that can serve as potential triggers of alloimmune responses even outside of mismatches at the highly polymorphic HLA locus. Several data have now interrogated non‐HLA mismatches in multi-ethnic and heterogenous renal allograft cohorts in a quantitative and genome‐wide basis (17–19). These data identified that global non‐HLA mismatch signals significantly associated with allograft rejection and/or survival. Hence, genome-wide dissimilarity between D–Rs or increasing genome-wide D–R “mismatches” have consistently emerged a clear predictor of graft outcomes, independent of HLA (17, 18, 20). These genome–genome interactions with increasing mismatches are reported to relay donor–recipient peptide differences outside of HLA but still conform to the traditional ‘missense hypothesis’ paradigm (17, 21). Here, non-HLA antigens involved are products of allograft-expressed donor genes that carry non-synonymous single nucleotide polymorphisms (nsSNPs) generating polymorphic peptides that are recognized as non-self by the immune recognition apparatus of the recipient and trigger an alloimmune response.

**Figure 1:**
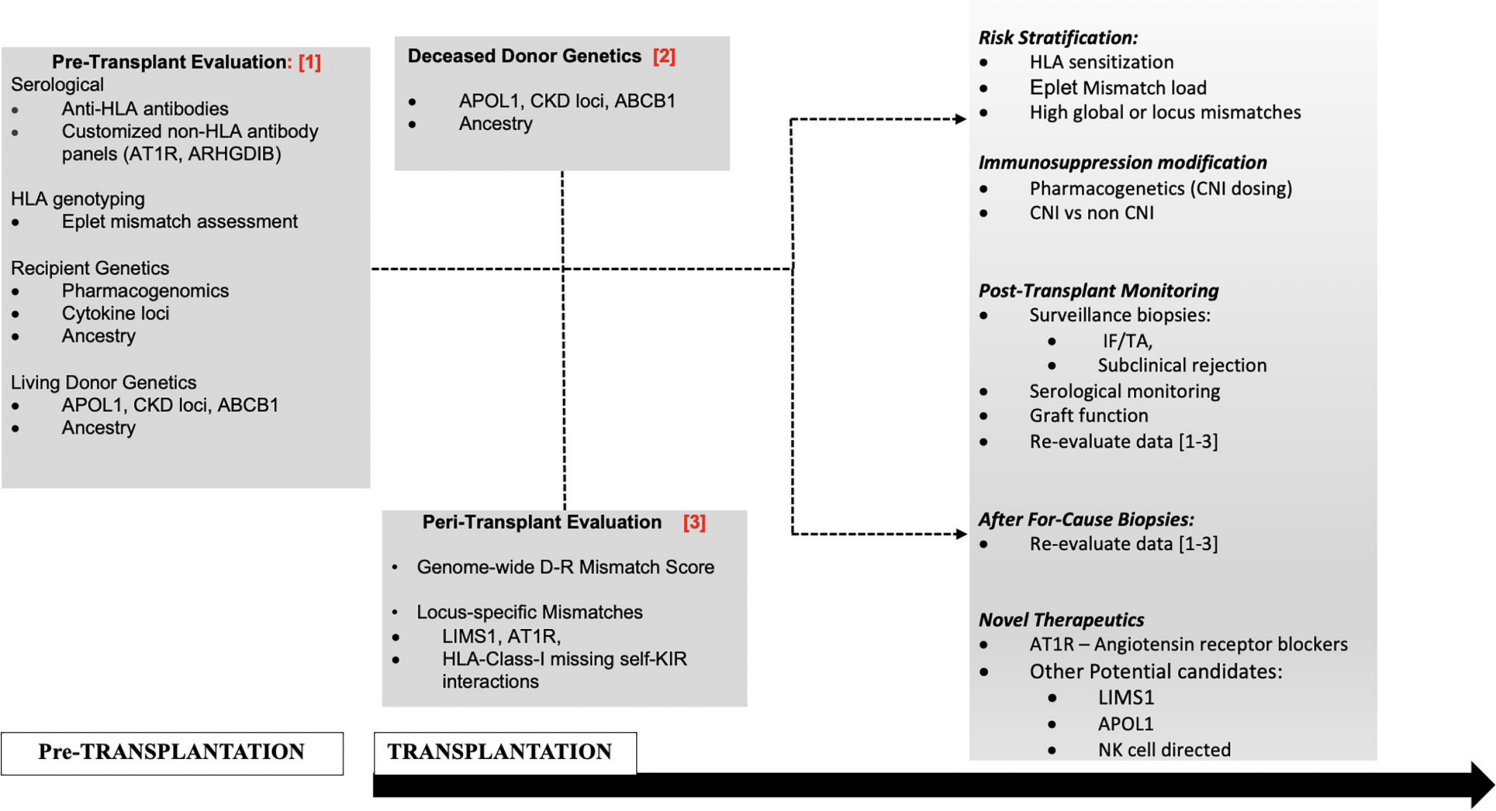
Proposed management schema to incorporate non-HLA genetic assessments in D–Rs.

However, novel mechanisms have also been invoked, without predicted donor-recipient (D–R) peptide dissimilarity or polymorphic peptide production (19, 22). First, specific genetic variants within donor or recipient genomes, are reported to induce qualitative or quantitative traits within the donor allograft or the recipient immune cells, regardless of interaction with the second genome during transplantation (15, 16, 18, 22–28). Furthermore, self-reported race in epidemiologic data (23, 29, 30), and donor- or recipient-genetic-ancestry in recent data continue to associate with outcomes through unclear mechanisms (16, 18). For instance, we used 1,000-genome data to project donor- and recipient-genetic ancestry onto a two-dimensional space allowing ancestry to be expressed as quantitative variables (proportions of African ancestry and/or Caucasian ancestry). We then identified that recipient genetic ancestry expressed as a proportion of African ancestry associated linearly with early creatinine trajectory up to two years (18). Since recipients are dependent on transplanted kidneys for creatinine excretion, this association could suggest altered creatinine generation or other mechanisms based on ancestry. In addition, human genome variation maps typically demonstrate near 3.5 million common- and 10 million-rare polymorphic loci between two unrelated individuals of European and African ancestries, i.e., D–R pairs of different ancestries are genetically further apart than D–Rs of similar ancestry with reference to non-HLA regions (31). Additionally, some genetic loci influencing graft survival are only relevant in specific ancestral backgrounds—for instance exonic variants in APOL1 in African ancestry- or African-Admixed genomes. Hence, a thorough analysis of D–R variants implicated in renal allograft outcomes will require giving consideration to donor variants, recipient variants (including ancestry-specific variants) and integrating information from D–R genome interactions or “mismatches” (**Figure 1**). Two research groups have now proposed comprehensive and integrative genetic approaches (20, 32) to simultaneously account for individual variants in recipients and also genome–genome interactions; however, such data are yet to be reported from actual patient cohorts.

Targeted analyses have primarily identified SNPs as associated with predefined phenotypes (e.g., acute allograft rejection or graft survival) (14, 33). Recent advances in genomic technologies and initiatives such as the HapMap Project have led to an increase in genome-wide association studies that use arrays that allow rapid identification of hundreds of thousands single nucleotide polymorphisms (SNPs) and copy number variants (CNV) across the human genome. Unbiased examinations of non‐HLA genomic sequence variations *via* genome‐wide association studies (GWAS) in D–Rs have thus associated novel genetic loci with graft outcomes (34–37). These donor–recipient GWAS studies have been mostly unsuccessful in identifying significant loci reproducibly. Only one of these three studies simultaneously considered both donor and recipient genomic variants. To improve the yield of GWAS data in transplantation, transplant-specific SNP-arrays have been developed enriched for non-HLA variants of specific relevance to transplantation and immune-mediated allograft outcomes (38). Besides SNPs, the relevance of interindividual non‐HLA variations from CNVs that may span exons or entire genes has been reported previously in bone marrow transplantation (BMT) (39). This is demonstrated by genes that show marked inter-individual CNVs, such as the Killer cell immunoglobulin-like receptors genes (KIRs). Duplication of gene sequences could lead to excessive or defective production of certain peptide sequences, contributing to peptide variation and serving to induce alloimmunity (40). Recent work has also reported a role for CNV-tagging SNPs in renal allograft rejection (22).

Non-HLA genomic variants identified from either targeted or unbiased analyses can be classified as *exonic*, *intronic*, or *intergenic*. Exonic variations in non-HLA regions may be non-synonymous or missense changes altering the resultant amino-acid, and in turn impacting protein structure/function and potentially conferring unique biologic properties. Here, alternate proteins coded by donor variants could promote anti donor non-self, classical immune responses. However, these loss- or gain-of-function variants could impact donor renal cell structure/function or in recipients, modulate immune responses, *without directly altering immunogenicity of the donor organ*. Non-coding variants may affect regulation of gene expression without impacting protein sequences (expression quantitative trait loci or eQTL) or alter gene splicing. Indeed, regulatory non-coding variants could themselves also induce loss or gain-of function. Consistently, a summary of GWAS reported that near 80% of GWAS-identified loci for complex traits localize to noncoding regions (41). In this review we summarize recent translational work on non-HLA variants and graft outcomes but focus on data describing non-HLA mismatches.

## Non-HLA Variants in the Donor and Allograft Outcomes

GWAS and targeted-gene analyses studies in kidney donors have thus far been limited, as donor DNA is often unavailable for retrospective analysis. **Table 1** summarizes important data regarding donor variants and outcomes. An analysis of both deceased and living donor samples in multiple cohorts has revealed that polymorphisms of 3 genes are likely important for allograft outcomes: APOL1, SHROOM3, and ABCB1.

**Table 1:** Donor variants and allograft outcomes.

Gene	Locus	Variant type^#^	Sample Size	Affected Outcome	Median Follow-Up (months)	Hazard Ratio (95% CI)
APOL1	rs73885319/rs60910145 (G1 allele) *rs143830837 (G2-allele)	Exonic, Syn Missense Exonic, Syn 6-bp deletion	1,158	Allograft survival	36	2.05 (1.39–3.02) (25)
SHROOM3	rs17319721 Locus (A allele)	Intronic Regulatory	203	12-month CADI-score**, eGFR	12	1.98 (1.10–3.59) (15)
CAV1	rs4730751 (AA genotype)	Exonic, Syn	785 (T)	Allograft survival	81	1.97 (1.29–3.16) (42)
			697 (V)		69	1.56 (1.07–2.27) (42)
UMOD	rs12917707 (T allele)	Intronic, Regulatory	393	Allograft survival	103.2	0.67 (0.46–0.97) (43)
ABCB1	​​rs1045642 (CC genotype) rs1045642 (T allele)	Exonic, Syn Exonic, Syn	189	eGFR	6,108	Independent association with eGFR (P = 0.02) (44) HR 1.32 (P = 0.04) (45)
			1,233	Time to allograft failure		

D–R, Donor–recipient; T, training set; V, validation set. *Alternate nomenclature rs71785313. ^#^Syn, Synonymous; CADI, Chronic allograft damage index; **based on Banff (2009).

### APOL1

High risk APOL1 genotypes (exonic G1 & G2 variants) have been recognized as an important predictor of chronic kidney disease and end stage kidney disease in individuals of African ancestry (46). Kidney transplantation has serendipitously served as a template to examine the association of APOL1-high risk mutations with kidney disease. Data sequentially reported with over 1,000 kidney transplant deceased donor recipients have demonstrated that the presence of 2 high-risk APOL1 alleles in the donor associates with reduced death-censored allograft survival (DCAL). More recently, in a cohort of 38 kidney transplant recipients with *de novo* collapsing focal segmental glomerulosclerosis, high-risk APOL1 genotypes were associated with poorer overall allograft outcomes (47). Thus APOL1 high risk gene polymorphisms likely confer structural abnormalities that predispose both native and donor kidneys to injury. APOL1 high risk variants have a prevalence of around 13% among African American ethnicity and genotyping of donors in the future may have implications for both recipients and living donors with the APOL1 high risk genotype (23). An ongoing concerted effort under the National Institutes of Health led by the National Institute of Diabetes and Digestive Kidney Diseases (NIDDK), partnering with the National Institute of Allergy and Infectious Disease (NIAID) & National Institute on Minority Health and Health Disparities (NIMHD) agencies is the “APOL1 Long-Term Kidney Transplantation Outcomes Network (APOLLO) study” (48). Since almost 50% of high-risk donor genotype APOL1 kidneys remain functional at 5 years (24), the APOLLO study will also use collected data to understand gene–gene and gene–environment interactions or second-hits that determine ultimate phenotype and allograft outcome.

### ABCB1

Donor ABCB1 polymorphisms have been associated with long-term kidney function among kidney transplant recipients who received CNI-based regimen (28). A GWAS of 189 transplants found that donor CC genotype (rs1045642) was associated with loss of allograft function (44). Other data have found similar associations between other donor ABCB1 polymorphisms and allograft outcomes in kidney transplants from American, Spanish, and Belgian and Chinese cohorts (28, 45, 49, 50). ABCB1 encodes an efflux transporter P-glycoprotein which plays an important role in the transport of calcineurin inhibitors (CNI) within enterocytes, hepatocytes, and kidney cells. Thus, it is possible that lower concentrations of the transporter may lead to higher intratubular CNI concentrations in kidney tubular epithelial cells and ultimately contribute to CNI toxicity and allograft loss. Whether the targeting of tacrolimus trough concentrations based on donor genotyping of ABCB1 improves allograft outcomes and the development of CNI toxicity remains to be seen.

### SHROOM3

In GWAS efforts to identify susceptibility loci for chronic kidney disease (CKD), loci in SHROOM3 have repeatedly emerged as associated with CKD using either cystatin or creatinine-based equations, especially in Caucasian-predominant cohorts. In a prospective cohort of kidney transplants, we studied the impact of the top ranked Shroom3 locus from CKD-GWAS, rs17319721, on allograft histology and survival. Shroom3 is an actin binding protein with roles in epithelial morphogenesis *via* recruitment of rho kinases to facilitate apical myosin contraction (51, 52). We showed that the CKD-associated A-allele at this locus was a TCF7L2-beta Catenin binding cis-eQTL for kidney Shroom3 expression. Increased allograft expression of Shroom3, as well as 1- or 2-copies of the donor A-allele (in a dominant model) (53) associated with increased fibrosis by 12-month post-transplant and reduced 36-month allograft survival (15). The recipient genotype did not impact histology or function. Mechanistically, Shroom3 overexpression promoted profibrotic TGF-beta signaling in tubular cells, while tubular-specific shroom3 knockdown reduced fibrosis in a murine model. In our data, recipients of live-donor A-allele allografts had the highest relative risk for increase in 12-month fibrosis scores. An independent report among recipients of LD allografts similarly showed an adverse impact of A-allele organs on 6-month GFR (44). In this case, the presence of ABCB1-risk variants interacted with the association of A-allele and lower EGFR.

Intriguingly, the same Shroom3 locus was also associated with reduced albuminuria in CKD populations (54). A similar protective impact on albuminuria was identified in our analysis in recipients of allografts homozygous for the A-allele in a recessive model for analysis (53). These data point to a complex effect of this enhancer rs17319721 locus and Shroom3 protein, in albuminuria and renal fibrosis, data that need further mechanistic and larger scale human studies.

Other donor Variants: Caveolin-1 (CAV1) is the primary component of caveolae, membrane invaginations that are abundant in endothelial cells, promote cellular transport and regulate signal transduction (55). An intronic CAV1 variant, rs4730751, when present in the donor was shown to increase risk of allograft failure in US and Irish cohorts (42). Data from the Gene Tissue Expression (GTEX) database suggests that this eQTL variant effects CAV1 and CAV2 expression in cell lines. However, a subsequent US study did not reproduce this association (45). In GWAS, variants in uromodulin (UMOD), specifically T-allele at rs12917707, have repeatedly been associated with CKD (56, 57). Elegant mechanistic data have shown eQTL function of this intronic variant mediating a protective role in salt sensitive hypertension by altering UMOD levels (58). A recent report showed a marginal protective effect of the donor T-allele at this locus on graft survival in Caucasian recipients (43).

## Non HLA Variants in Recipient Genome Associated With Renal Allograft Outcomes

Several excellent reviews have tabulated associations of donor and recipient SNPs with allograft outcomes (10, 59, 60). Most previously identified loci in targeted analyses are in genes with an immunomodulatory role, or in proteins involved in drug metabolism pathways (61–63). These variants are summarized in **Table 2** along with recipient only GWAS studies.

**Table 2:** Recipient variants and allograft outcomes.

Protein	Allele/Gene polymorphism	Outcome	Median follow up (years)	Sample size (N)	Results	Reference
TNFA	TNFA promoter-308 G A (high producer genotype)	Rejection episodes, Rejection severity and steroid responsiveness	5	100	TNFA high producer genotype was associated with multiple rejection episodes (p = 0.0047), TNFA high producer associated with severe and steroid resistant rejection episodes (p = 0.025)	Sankaran et al. (10)
IFNG	IFNG + 874 Homozygous T/T (high IFNG level)	Acute rejection, Chronic allograft nephropathy (Banff, 1997)	1	74	Increased T/T genotype among rejection group (p = 0.0061), Increased T/T genotype among those with chronic allograft nephropathy (p = 0.0067)	Crispim et al. (61)
FOXP3	FOXP3 promoter region (GT)n dinucleotide repeat S-Genotype (SG) (≤ (GT)15 & SL, S/SS)	Acute rejection, DCAL	7.7	599	SG superior in graft survival censored for death (logrank, p= 0.013), Advantage of SG in graft survival HR 0.67 95% CI 0.48–0.94 (p = 0.02)	Engela et al. (62)
CYP3A	CYP 3A5 CYP3A5*1/*1	Tacrolimus dose, Tacrolimus concentration-to-dose ratio	1 month	80	Significantly associated with daily tacrolimus dose, 1 month after tacrolimus treatment with gene-dose effect (p = 0.05), Mean concentration:dose ratio was lower (p = 0.01)	Thervet et al. (63)
–	rs3811321 (Chr14), rs6565887 (Chr 18)	Cr at 5 years, Long-term allograft function	10	326	Both variants predicted long term allograft function, p = 0.04	O’Brien et al. (35)
–	rs3811321 (Chr 14), rs6565887 (Chr 18)	Primary endpoint: DCAL, Secondary endpoint: All-cause mortality	7.6	1638	No association of variants to either end-point HR 0.88, 95% CI 0.62–1.25, p = 0.48, HR 0.87, 95% CI 0.59–1.29, p = 0.50	Pihlstrøm et al. (64)
PTPROCCDC67	rs10765602, rs7976329	TCMR occurring in the first year after renal transplantation (Banff, 2007)	1	778	Significant association of both loci with outcome (p = 0.02 and 0.01)	Ghisdal et al. (36)
LIMS1	rs893403	Risk of rejection with homozygous presence of allele in recipient	8.6	705	Homozygous genotype for deletion-tagging allele at higher risk of rejection HR 1.84 (1.35–2.50), p = 9.8 × 10^−5^	Steers et al. (22)
LIMS1	rs893403	Primary outcome: TCMR, ABMR (Banff, 2013) Secondary outcome: Allograft survival	11.4	841	Homozygous genotype associated with higher risk of TCMR HR 2.43 (1.44–4.12), P = 0.001	Caliskan et al. (26)
APOL1	G1,G2 alleles	Long-term allograft outcomes in recipients homozygous for high-risk alleles	5	119	No difference in allograft survival at 5 years for recipients with high-risk APOL1 genotypes (HR 0.96, 95% CI 0.61–1.49, p = 0.840)	Lee et al. (65)
APOL1	G1,G2 alleles	TCMR (Banff, 2009) DCAL	2	507	Number of recipient APOL1 risk alleles is associated with increased risk of TCMR and increased DCAL, HR = 2.14 per additional copy of risk alleles	Zhang et al. (16)

DCAL, death-censored allograft loss; Chr, chromosome; TCMR, T-cell mediated rejection; Cr, creatinine.

CYP3A5*1/*1 = homozygous expression of allele linked to high expression of CYP3A5.

In the first recipient-only GWAS of 326 patients, O’Brien et al. were unable to find any loci of genome-wide significance but did find associations between SNPs on chromosomes 14 and 18 with medium-term serum creatinine and long-term graft survival. However, these findings failed to be validated by a subsequent study (64).

Another recipient-only study (36) looked at biopsy-proven acute T-cell mediated rejection occurring in the first year post-transplantation and identified 5 loci of significance of which two remained significantly associated with acute rejection in univariate and multi-variate analysis in the replication cohort—PTPRO which plays several roles at the immune synapse and B-cell receptor signaling and CCDC67 which is a ciliary gene.

Steers et al. in their investigation of the concept of ‘genomic collision’ identified the LIMS1 locus as associated with allograft rejection in a time-to-event analyses. Genomic collision refers to a loss of function variation in nonessential genes in the recipient that triggers an alloimmune response to the normal variant expressed in the donor leading to poor allograft outcomes. In the innovative approach used in the first phase of their study, they focused on CNV-tagging SNPs that would associate with loss or reduced copy-numbers when homozygously inherited by the recipient (22). In their discovery cohort (n = 705), 50 deletion CNV-tagging polymorphisms were tested for association with biopsy-proven rejection. Here, a homozygous variant at the chromosome 2q12.3 locus within the LIMS1 gene associated independently with rejection (G/G variant at this locus), HR 1.84, P = 9.8 × 10^−5^. In three large validation cohorts (n = 2,004), recipients who were homozygous for the deletion-tagging allele (G/G) had an 84% higher risk of rejection than A/A or A/G. Functionally, the rs893403-G risk allele associated with lower LIMS1-mRNA expression in renal tubulo-interstitium. LIMS1 is a cytoplasmic protein normally expressed in the human distal nephron and endothelium and with a role in cell-adhesion and integrin signaling. Interestingly, LIMS1 expression was further induced by ischemia with appearance of cell-surface LIMS1. The authors then demonstrated the presence of anti-LIMS1 IgG in a subgroup of high-risk D–R pairs with AR.

A subsequent study by Caliskan et al. (841 recipients) examined LIMS1 rs893403- risk alleles in a single center study for the outcome of any rejection (26). Using a median of 11-year follow-up time, they identified significantly higher rate of T-cell mediated rejection in recipients with the GG genotype (OR = 2.4) vs A/G or A/A. This study, however, was unable to demonstrate an association with antibody mediated rejection (the proposed mechanism from prior data) or with 10-year allograft outcomes in the GG versus AG/AA groups. From these studies, while the association of rs893403-risk allele in recipients with adverse allograft outcomes was consistent, the mechanism requires further understanding.

As discussed above, the presence of APOL1 risk variants in the donor and association with outcomes in well-established. However, Lee et al. were the first group to examine the presence of APOL1 risk alleles in African American recipients of kidney transplants and their impact on transplant outcomes. In their cohort of 119 patients, 48% were carriers of two APOL1 risk alleles but no association was found between the presence of any number of risk alleles and allograft loss or death-censored allograft loss (65). The donor APOL1 genotypes were not identified in this study and the study reported an unusually high rate of death-censored allograft loss of about 25% at 5 years, likely affecting the suitability of this dataset to evaluate impact of recipient APOL1variant associations.

In contrast, in a recent work, we re-evaluated the role of APOL1 variants in the recipient in determining allograft outcomes (16). Two transplant cohorts were studied, the Genomics of Chronic Allograft Rejection (GoCAR) and the Clinical Trials in Organ Transplantation (CTOT) and an association was found between the presence of APOL1 risk alleles and an increased risk of acute T-cell mediated rejection, and reduced long-term allograft survival. In these data, we did not find association with recipient survival. Interestingly, the number of APOL1 risk alleles associated with T-cell mediated rejection in an additive model (distinct from the recessive model observed with donor APOL1 variants) and was independent of genetic recipient ancestry. *Ex vivo* transcriptome data obtained from peripheral blood mononuclear cells (PBMCs) pre-transplant and from healthy controls, showed a clear signature of immune activation in CD4, CD8, and NK cells in patients with APOL1 G1 or G2 variants vs G0, implicating APOL1 risk alleles with immune activation in these cells. These provocative data will be examined in the ongoing nation-wide APOLLO study and lay the ground for mechanistic work to understand the immunomodulatory role of APOL1.

## Donor–Recipient Mismatches

As observed in the data described above, several donor-only or recipient-only GWAS reported either lack of discovery of loci of genome-wide significance or, lack of independent validation of previously identified loci. Several important reasons exist for these predominantly negative findings, namely, the lack of large sample sizes, heterogeneity of phenotypes tested (for instance clinical vs subclinical rejection), and insufficient accounting for baseline ancestry-based variation. In addition, transplantation involves an interaction of two independent genomes, and resulting mismatches have complex mechanisms and potentially could have larger effect sizes than conferred by donor- or recipient variants alone. In the next section, we discuss locus-specific mismatches (potentially signaled) by the development of unique non-HLA antibodies, and genome-wide mismatch data in kidney transplantation.

## Locus-Specific Mismatches and Non-HLA Antibodies

### MICA

Major histocompatibility class I-related chain A (MICA) is a surface glycoprotein expressed on endothelial cells, epithelial cells, fibroblasts and other cells, but not expressed on peripheral blood lymphocytes. Following transplantation, exposure to allogeneic MICA can result in formation of antibodies. These antibodies would not be detected by traditional cross-matching techniques, as they are not expressed on peripheral-blood lymphocytes. It was proposed that these antibodies would contribute to allograft rejection as MICA antigens expressed on endothelial cells can be cytotoxic in the presence of serum complements.

A study by Zou et al. included 1,910 kidney transplant recipients who received deceased donor kidneys and measured IgG against MICA antigens (66). Of the 1,910 recipients, 217 were found to have antibodies against MICA alleles. There was an increased rate of allograft rejection among those with anti-MICA antibodies. 1-year graft-survival for those with anti-MICA antibodies was 88.3 ∓ 2.2% compared with 93 ∓ 0.6% in those that were negative for the antibodies. Interestingly, 37 patients had both anti-MICA antibodies and anti-HLA Class I antibodies, and 35 patients had anti-MICA antibodies and anti-HLA Class II antibodies. However, it was noted that anti-MICA antibodies correlated with poor outcomes in those who were not HLA sensitized. The inciting event for anti-MICA antibody production is suspected to be cross-reactivity with an environmental substance. Unlike HLA antibodies, this study suggests anti-MICA antibodies were not higher in those who received more blood transfusions.

### Vimentin

Antigens expressed on endothelial cells, could be considered to be the “first responders” in the setting of inflammation and ischemia-reperfusion injury. Vimentin is an intermediate-filament protein expressed by endothelial cells and regulates cellular cytoskeletal structure, cell signaling and proliferation. In both heart and kidney transplant recipients, anti-endothelial cell antibodies have been identified, including anti-vimentin antibodies. Anti-vimentin antibodies (AVA) may be produced after tissue injury or infections, situations wherein vimentin is expressed on cell surfaces and “seen” by the immune system (67). In these cases, the development of AVA is a part of autoimmunity and the presence of these AVA pre transplantation are associated with chronic allograft injury, both in heart and kidney transplant recipients (68). Elevated levels of these antibodies along with the presence of C4D staining on biopsy also correlated with interstitial fibrosis and tubular atrophy. Divanyan et al. showed that AVA concentration (pre-transplant) greater than 15 μg/ml was associated with two-fold higher risk of early IF/TA (67). However, a study in rats after renal transplantation observed *de novo* development of AVA after transplantation suggesting either auto-immune or allo-immune mechanisms for AVA generation.

## Anti-Endothelial Cell Antibodies

Delville et al. evaluated kidney transplant recipients with acute microvascular rejection (AMVR) within 3 months post-transplantation who did not have anti-HLA DSA identified (on Luminex single antigen bead assay and biopsy) (69). They measured serum levels of known anti-endothelial cell antibodies (AECAs)-anti AT1R, anti-endothelin-1 type A (ETAR), IgG natural polyreactive antibody (NAb)-on the day of transplantation. They had histologic controls (recipients diagnosed with AMR with identified anti-HLA DSA) and biologic controls (recipients with stable graft function for 1 year). In comparison with histologic controls, recipients without identifiable anti-HLA DSA had more severe endothelial/vascular injury with vasculitis and thrombotic microangiopathy. In comparison to biologic controls, they found no significant difference in the AT1R or ETAR, although patients with AMVR had a higher likelihood of a positive endothelial cell crossmatch, suggesting antibodies against diverse endothelial antigens. However, it is notable that there was a strong correlation between anti-AT1R and anti-ETAR, again lending credence to the hypothesis that some patients have a broad autoimmune response.

### LG3 and PERLECAN

Perlecan is a vascular basement membrane proteoglycan that helps maintain endothelial integrity. Pathogenic antibodies targeting Perlecan were eluted from a Fisher-to-Lewis rat transplantation model (70). Subsequent data suggests that endothelial cell injury and apoptosis results in release of a C-terminal fragment from Perlecan, Laminin G-like domain 3 (LG3). In 2012, Soulez, Hebert et al., found that pre- and post-transplant anti-LG3 levels were elevated in kidney transplant recipients with acute vascular rejection (compared to recipients with tubulointerstitial rejection and those with normal graft function) (68, 71). The effect of anti-LG3 antibodies were studied in an animal model of vascular rejection (using orthotopic aortic transplantation in fully mismatched MHC mice). Murine anti-LG3 IgG or control IgG were passively transferred to the mice. Increased neointima formation, C4D deposition and allograft inflammation were noted in recipients of aortic allograft transferred with anti-LG3 (71). Subsequent reports have associated pre-transplant anti-LG3 levels with early outcomes in kidney (72) and Liver transplantation (73). Thus, elevated levels of anti-LG3 are associated with vascular rejection and antibodies to LG3 contribute to allograft vascular injury.

### ETAR

Endothelin-1 type A receptors (ETAR) are G-protein coupled receptors that are expressed widely in the human body, including renal vascular smooth muscles. Activation of ETAR by endothelin 1 regulates blood flow *via* vasoconstriction or vasodilation. Binding of ETAR by antibodies to ETAR (anti-ETAR) results in sustained activation (74, 75). Allograft function at one-year post-transplant in recipients with pre-transplant anti-ETAR was reported to be worse, compared to those without anti-ETAR detected in a subsequent study. In another study, mild to severe intimal arteritis was seen more often in recipients with anti-ETAR antibodies (76). In pediatric kidney transplant recipients, anti-ETAR at any time (pre- or post-) was strongly associated with anti-AT1R and those with both antibodies had more arteritis, elevated IL-8, and decline in kidney function (but not rejection or allograft loss) (77).

### ARHGDIB

Kamburova et al. assessed the presence of 14 non-HLA antibodies with allograft survival using a customized non-HLA antibody assay in a nationwide dataset of 4,770 recipients. In recipients of deceased donor kidneys, they found presence of autoantibodies to Rho GDP-dissociation inhibitor 2 (ARHGDIB) was associated with graft loss. They also found a 13% lower death-censored graft survival at 10-years post deceased donor transplants among recipients with these antibodies, independent of anti-HLA antibodies (78). The association between AMR and pre-transplant anti-ARHGDIB was identified in a subsequent study of 203 patients (79). Pre transplant anti-ARHGDIB and AMR were risks for allograft failure independent of anti-HLA DSA, and histologic AMR in these cases was associated with increased expression of intrarenal ARHGDIB gene. The results were notable for the synergic effect of pre-transplant autoantibodies to ARHGDIB and DSA on risk of graft failure. Interestingly, pre transplant anti-ETAR levels were not associated with graft survival in these data. In 2020, Betjes and colleagues studied patients with chronic active AMR (caAMR) and compared them to a group with no rejection. Fourteen non-HLA antibodies were evaluated pre-transplant and at the time of biopsy of which, antibodies to ARHGDIB were significantly higher in those with caAMR compared to the group without rejection. In this study however, the presence of pre transplant anti-ARHGDIB antibodies did not associate with cAMR diagnosis, and the post-transplant levels did not associate with graft loss, contrary to prior data (80).

Consistent across these and mechanistic data (81) is the need for pre-transplant generation of anti-LG3, anti-ETAR or anti-ARHGDIB either as auto-immune phenomena or as a reflection of overall sensitization status (79, 82); hence, a clear association with D–R dissimilarity, allo-immunity and these antibodies has not yet been uncovered.

### AT1R

In 2005, Dragun et al. studied 33 patients with steroid-refractory acute allograft rejection in a landmark study (83). They evaluated the serum of the patients for the presence of donor-specific anti-HLA antibodies (DSA) and also antibodies against angiotensin II type 1 (AT1) receptor, and allograft biopsies for C4d and tissue factor. Thirteen patients were found to have DSA, 16 patients did not have DSA but had malignant hypertension, while the remaining 4 had no DSA or malignant hypertension. They found that all 16 patients with malignant hypertension (a vascular rejection phenotype) were positive for antibodies against AT1R and had a worse allograft survival. The authors demonstrated the binding of anti-AT1R to AT1R resulted in activation of ERK1/2 signaling cascade, similar to angiotensin II itself. Furthermore, the use of antibody removal and blockade of AT1R with losartan, as part of management of these patients significantly improved allograft survival compared to those receiving only standard rejection treatment. Since this original report, multiple datasets (>100 publications) have evaluated the association of ant-AT1R antibodies and allograft outcomes—elegantly reviewed in (84). Some important themes can be distilled from these reports.

First, the association with graft survival of anti-AT1R levels even before transplantation is demonstrable (85), albeit with few exceptions (79), possibly implying auto-immunity. An association between anti-AT1R, and other non-HLA-antibodies (ETAR), and synergistic impact on graft outcomes in the presence of anti-HLA-DSA is reiterated (77, 84). For instance, Crespo et al. retrospectively analyzed 118 kidney transplant recipients with allograft biopsies. Pre- and post-transplant serum levels of the following were checked: HLA DSA and specific non-HLA abs [MICA, AT1R, ETAR, crossmatches with primary aortic endothelial cells (ECXM)]. Of 118 participants, 52 had AMR, 14 had IFTA, while 19 had normal histology. Here, pre-transplant HLA-DSA and anti-AT1R were more frequent in those with AMR compared with IFTA and normal histology (86). In 1,845 kidney transplant recipients, with anti-AT1R and DSAs evaluated during their first rejection episode or at 1-year post-transplantation, the presence of anti-AT1R was associated with higher incidence of AMR at 1-year post-transplant and increased risk of allograft loss, seen in strata of patients with/without DSAs. These data suggest synergistic effects of the HLA and non-HLA (AT1R) antibodies.

Next, an extension of anti-AT1R mediated injury to non-kidney organs and a more generalized role for this autoantigen in allo-transplantation is also identifiable (87, 88). However, aside from autoimmunity, in our work, we identified that increased genome-wide mismatch was proportional to AT1R-locus mismatches. Within a subgroup of Caucasian-to-Caucasian transplants, such genome-wide mismatches, associated with an increased risk of AT1R antibodies by 2-year post-transplant, implicating AT1R locus mismatches in the development of AT1R antibodies (18). These data need validation from larger cohorts with corresponding pre-transplant AT1R level comparison.

Recent data has described the “classical” phenotype of anti-AT1R associated AMR, with a higher prevalence of hypertension, more vascular injury and arterial inflammation, higher levels of endothelial-associated transcripts, and lack of complement deposition in allograft capillaries (89). Indeed, the expression of AT1R in allograft biopsies together with the presence of antibodies to AT1R was shown as a specific risk factor for graft loss (90). Finally, the role of these antibodies, the unique signaling mechanism identified and responsiveness to a non- immunosuppressive agent (Losartan) has opened the field up to discovery and phenotyping of non-HLA antibodies and genetic variants.

### LIMS1

In the Steers et al. paper, the signal for increased risk of TCMR was enriched when donors with A/A or A/G introduced into G/G recipients were considered vs all other G/G recipients. These findings were validated across 3 cohorts where donor genotype was available. This intriguing finding suggested that while the risk of increased rejection may primarily travel with having G/G genotype in recipients, transplantation of A-allele donor kidneys made this risk manifest for unclear reasons. Furthermore, these data suggested a “directionality” for rs893403 mismatch which associated with worse outcomes when A-allele was introduced into G/G recipients, and not vice versa. Within a subgroup of this cohort, the authors identified the presence of anti-LIMS1 antibodies in rejection cases with high-risk direction mismatch only, suggesting that a classical adaptive immune response is involved in these findings. Since the intronic rs893403 variant does not induce a polymorphic or generate dissimilar peptides, the overall mechanism underlying these exciting findings needs further definition.

It must be observed that while most of these studies demonstrate associations of specific non-HLA antibodies with rejection or graft survival, mechanistic data testing causation in animal models are limited. Further, current commercial Luminex or ELISA panels for these targets have been used only in research settings and are not licensed for use in clinical practice. AT1R assays are exceptional in this regard and frequently utilized in clinical practice.

### KIR-Locus Mismatches, NK-Cells and “Missing-Self”

Recent studies have challenged the dogma that the presence of microvascular inflammation (MVI) during graft rejection is the histologic hallmark of antibody mediated injury. Using 129 kidney transplant recipients, Koenig et al. demonstrated that histologic evidence of microvascular inflammation was noted independent of the presence of circulating antibodies in half of the cases (91). They postulated that this may be explained by NK cell mediated activation as a consequence of missing self (MS). NK cells are educated to recognize “self” HLA class-I molecules *via* Killer cell immunoglobulin-like receptors (KIRs), which deliver inhibitory signals to NK cells preventing NK-activation and injury (40). KIRs and HLA Class-I molecules suggest co-evolution, and extensive inter-individual KIR variability is contributed to by CNVs as well as alleleic forms (92). KIRs are activated by activating KIRs in the absence of inhibitory signals i.e., “missing-self” (MS) responses, when they interact with specific class-I HLA molecules i.e., non-self (93). In such mismatched donor HLA-with-recipient KIR combinations, donor endothelial cells would be unable to deliver inhibitory signals to recipient NK cells, causing NK cell activation, endothelial cell damage and MVI. To confirm the hypothesis of NK-cell mediated missing-self responses, they performed high-resolution genetic analyses of recipient inhibitory KIRs and donor HLA class-I to identify situations of missing self and showed for the first time that recipients with antibody-independent MVI had significantly more genetically predicted missing-self than matched controls.

In another large study of 924 kidney transplant recipients (94), missing-self was identified in 399 of 924 transplantations with co-occurrence of two or more missing-self types in 110 transplants. The risk of MVI was significantly higher in D–Rs with two (HR 1.66) or three-MS types (HR 3.95) compared with no MS. While MS was significantly associated with increased incidence of MVI and development of transplant glomerulopathy, it was not significantly associated with graft survival (HR 1.23, P = 0.53).

The same group investigated whether missing self-amplified DSA-dependent NK cell activation in chronic AMR and explained some of the heterogeneity in AMR outcomes. In 135 kidney biopsies suggestive of complement-independent chronic AMR, those that had MS among D–Rs, had worse overall allograft outcomes (P = 0.02) (95). Complement independent mechanisms of antibody mediation cellular toxicity (ADCC) by innate immune cells (including NK-cells) depends on the interaction of Fc gamma receptors (FCGRs) with DSAs that are bound to endothelial cells but may not activate complement (96–98). An increased density of NK cells was found in microvascular and interstitial compartments in grafts from MVI+DSA+C3d−MS+ patients (36 ± 49, P = 0.03). Transcriptomic analysis also revealed more activated NK cells in the grafts of MVI+DSA+C3d−MS+ patients than in those of MVI+DSA+C3D−MS− which showed that MS also synergized with DSA to promote NK cell recruitment and activation during chronic AMR with worse allograft outcomes.

Transcriptomic signatures of renal allograft biopsies have also revealed enrichment of the FCGR3A (CD16) transcripts correlating with DSA and ABMR (99). In addition, increased expression of FCGR3A was seen on circulating NK cells of kidney transplant recipients with chronic AMR (100). Litjens et al. in a recent study investigated the role of the V/V genotype of the FCGR3A 158-F/V polymorphism in death censored allograft survival in chronic AMR (101). The authors looked at 133 patients with chronic AMR and found that the V/V-genotype was associated with a higher glomerulitis score and was an independent risk factor for DCAL with HR 1.98. The V/V genotype was also associated with increased NK cell CD 16 expression and function. This genotype was not, however, useful for predicting development of chronic AMR after kidney transplantation as allele frequency was the same in the control cohort of transplant recipients without a diagnosis of chronic AMR.

## Genome-Wide D–R Mismatches

The first ever genome-wide association study (GWAS) that combined the analysis of kidney donors and recipients was undertaken by the Wellcome Trust Case Control Consortium (WTCCC) (34). They analyzed 2,094 kidney donor–recipient pairs in the discovery and an additional 5,866 pairs in the replication phase. Several models were tested, including donor-only and recipient-only GWAS and donor–recipient interaction models and donor–recipient mismatches in copy number variants were also examined. The outcomes tested were time to acute rejection and graft failure. Unfortunately, the study was unable to find any statistically significant signals outside of the HLA system. This was attributed to heterogenous population phenotype and small study size, which was likely insufficient to detect complex genotypic interactions especially in the discovery cohort.

Although a negative study, the first donor–recipient GWAS highlighted the limitations of small study populations at single transplant sites and the complexity of donor and recipient covariates and disease-related phenotypes. As such, the International Genetics & Translational Research in Transplantation Network (iGeneTRAiN) consortium has been established to minimize limitations due to population size (102). This is a multi-site consortium that encompasses >45 genetic studies with ~51,210 solid-organ transplant subjects with existing genome-wide genotype data sets, designed and statistically powered to allow for a spectrum of analyses to be performed. This has laid the groundwork for multi-center GWAS studies since the design of the ‘TxArray’ (38), a customized genome-wide genotyping tool with tailored content to capture transplantation-related and variants.

Mesnard et al. devised an allogenomics mismatch score based on all possible cell surface antigen mismatches between D–R pairs based on amino-acid mismatches in transmembrane proteins (21). This strategy was based on immunologic and biologic plausibility since, cell surface expressed and/or secreted proteins are likely to be first encountered by the immune cells of the recipient, and non-HLA antibodies could directly interact with these proteins. They showed that in their study population of 53 D–R pairs, allogenomics mismatch score had a significant effect on eGFR independent of HLA matching, donor age and time since transplantation at 36 months. The results of the study were novel in that they suggested that the total burden of allogenomics mismatch might be more predictive of long-term allograft function rather than locus-specific mismatches as had been previously believed. This study, however, did not look at incidence of acute or chronic allograft rejection as an outcome of allogenomic mismatch.

Subsequently, Pineda et al. looked at focused non-HLA D–R mismatches *via* exome sequencing and outcomes of biopsy-proven rejection versus no rejection on 28 D–R pairs (19). They were able to identify 123 unique non-HLA variants that were nominally associated with antibody-mediated rejection, cell-mediated rejection and no rejection. Of these, 94 variants were found to be most enriched for AMR, 25 variants for CMR and 4 variants enriched for low immune risk and no rejection. The products of these genomic variants were found to be expressed on the kidney, blood vessels, immune cells, and involved in cell-surface expression. Interestingly, several identified variants were non-coding and localized to predicted regulatory regions within the respective genes. The authors proposed the addition of a minimal non-HLA variant list to current HLA testing to enhance ability to predict AMR and evaluate immunologic risk to the allograft.

Reindl-Schwaighofer and Heinzel et al. were the first group to show that genome-wide genetic incompatibility between kidney donors and recipients in a prospective cohort significantly associated with death-censored graft survival (17). They genotyped 477 pairs of kidney recipient and donor pairs with stable graft function at three months to look for genome-wide mismatches in non-synonynous single nucleotide polymorphisms (nsSNPs). They based their investigation on biological plausibility stratifying genome-wide mismatches as those predicted to induce donor-recipient peptide changes (i.e., nsSNPS) that were within transmembrane or secreted protein, adjusting for mismatches in all other loci and genes as covariates. Thus, they analyzed 59,268 nsSNPs and found that a median of 1892 nsSNP mismatches existed between donors and recipients. When adjusted for HLA eplet mismatches (not simply 2- or 4-digit HLA-mismatches), the extent of nsSNP mismatch was independently associated with graft loss. Moreover, using customized peptide arrays they were able to verify a donor-specific alloimmune response to genetically predicted mismatched epitopes in 16 of 25 patients with biopsy-proven chronic antibody mediated rejection. The pathogenic role of these specific antibodies is unclear and remains to be explored. Furthermore, while surface expressed protein mismatches are an attractive approach, this does not completely account for antigens utilizing the indirect pathway of antigen presentation or the surface expression of normally intracellular proteins after cytokine- or injury stimuli.

Using the multi-ethnic genomics of chronic allograft rejection (GoCAR) cohort, we identified a novel correlation between quantitatively estimated recipient ancestry estimated by ADMIXTURE, and post-transplant outcomes. To estimate genome-wide differences, specifically in intra-ancestry donor–recipient pairs, we utilized the proportion of genome-shared identity by descent variable (pIBD) measuring relatedness between any given D–R pairs in this cohort. We observed that this pIBD variable which included coding and non-coding variants significantly associated with long-term death censored graft loss (18). It must be noted that a subsequent dataset from the UK was unable to identify an association of pIBD score with graft loss, when adjusted for serologically obtained HLA-mismatches (103). Regardless, we intriguingly observed that pIBD inversely associated with early (<1 year) development of vascular intimal fibrosis (Banff Cv score) and interstitial fibrosis and tubular atrophy (IF/TA), even when adjusted for donor-derived intimal fibrosis in pre-perfusion biopsies. We know based on findings from large biopsy studies that (a) IF/TA of undetermined etiology is the only lesion seen in 30–40% of all allografts that fail (3, 104) and (b) IF/TA exhibits the same gene expression profile as that of T-cell mediated rejection even without active tissue inflammation (5, 105). The strength of this well-phenotyped prospective cohort was the uniform reporting of surveillance and indication biopsies by a central core lab, and the long-term graft loss data obtained from UNOS and ANZdata.

## Conclusion

The increasing focus towards improving long-term allograft outcomes has spurred investigations to understand the full extent of anti-donor responses that culminate in allograft injury and organ failure after transplantation. These efforts have expanded from an HLA-centric view to a broader examination of genetic variants in the donor and recipient genomes and “mismatches” thereof. These new and emerging data show a clear role for genome-wide mismatches (independent of the HLA region) in rejection and/or long-term renal allograft outcomes. However, there still remain questions regarding the application of these findings to clinical scenarios. Using the TRIPOD checklist benchmarks (106) for evaluation of multivariable prediction models, each currently available retrospective dataset only included a single development cohort, without internal validation strategies (17–19, 21), while one study used simulated samples (20). While the derived predictive variables in each of these data aimed at quantifying genome-wide mismatch signals, there was heterogeneity in methodology (sequencing vs SNP-genotyping), and analyses pipelines between these datasets. When studies aimed to test predictive variables derived from other datasets (103), these findings did not identically replicate in their predictive potential. Notably however, marked heterogeneity existed in patient characteristics between cohorts—geography (i.e., access to reimbursement for immunosuppression), follow-up duration, outcomes tested (ABMR, AR, survival), overall graft survival rate, and genetic/ancestral composition, and could explain these inconsistencies. Hence what remains to be better defined in ongoing studies are optimal strategies to best capture genome-wide differences for distinct outcomes whether based on ancestry, relatedness, or biological plausibility (transmembrane and secreted proteins alone, NK-cell KIR variation, regulatory regions, etc.), with the goal of minimizing the signal-to-noise ratio in genome-wide data. These approaches will likely need to be weighted for other cohort-specific predictors including donor- and recipient genetics. A highly significant application of this work is identifying individual non-HLA-genes, -genetic loci and/or locus mismatches of specific and disproportionate relevance to transplant outcomes. This could set the stage for risk-stratification and allocation based on these loci, with the potential for targeted therapeutics (after mechanisms are clearly established such as immunosuppression modification, plasma exchange for non-HLA antibodies or repurposed/novel therapeutics e.g., APOL1 (107). A potential management schema to incorporate non-HLA genetic assessments (as reviewed here) in D–Rs using a contemporary transplantation timeline is illustrated in **Figure 1**. Subsequently, determining validated non-HLA loci of interest in long-term allograft outcomes will help perform routine testing for variant mismatches to allow for precision-matching of organs in patients with multiple potential donors and provide personalized immunosuppression and surveillance goals for non-modifiable risk factors.

## Author Contributions

PJ and AR performed literature review and wrote the manuscript. LB reviewed and edited the section on non-HLA antibodies. MM was responsible for drafting, editing and organizing the manuscript. All authors listed have made a substantial, direct, and intellectual contribution to the work and approved it for publication.

## Funding

MM acknowledges funding from the NIH DK122164, and past pilot funding from the CTOT-19 study (PI: Peter Heeger; NIH U01AI063594) to study non-HLA donor-recipient genetic differences.

## Conflict of Interest

The authors declare that the research was conducted in the absence of any commercial or financial relationships that could be construed as a potential conflict of interest.

## Publisher’s Note

All claims expressed in this article are solely those of the authors and do not necessarily represent those of their affiliated organizations, or those of the publisher, the editors and the reviewers. Any product that may be evaluated in this article, or claim that may be made by its manufacturer, is not guaranteed or endorsed by the publisher.
